# Combination Across Domains: An MEG Investigation into the Relationship between Mathematical, Pictorial, and Linguistic Processing

**DOI:** 10.3389/fpsyg.2012.00583

**Published:** 2013-01-03

**Authors:** Douglas K. Bemis, Liina Pylkkänen

**Affiliations:** ^1^Department of Psychology, NYU-Abu Dhabi Institute, New York UniversityNew York, NY, USA; ^2^Department of Linguistics, NYU-Abu Dhabi Institute, New York UniversityNew York, NY, USA

**Keywords:** language, cognitive neuroscience, temporal lobe, domain-generality, combinatorics

## Abstract

Debates surrounding the evolution of language often hinge upon its relationship to cognition more generally and many investigations have attempted to demark the boundary between the two. Though results from these studies suggest that language may recruit domain-general mechanisms during certain types of complex processing, the domain-generality of basic combinatorial mechanisms that lie at the core of linguistic processing is still unknown. Our previous work (Bemis and Pylkkänen, 2011, 2012) used magnetoencephalography to isolate neural activity associated with the simple composition of an adjective and a noun (“red boat”) and found increased activity during this processing localized to the left anterior temporal lobe (lATL), ventro-medial prefrontal cortex (vmPFC), and left angular gyrus (lAG). The present study explores the domain-generality of these effects and their associated combinatorial mechanisms through two parallel non-linguistic combinatorial tasks designed to be as minimal and natural as the linguistic paradigm. In the first task, we used pictures of colored shapes to elicit combinatorial conceptual processing similar to that evoked by the linguistic expressions and find increased activity again localized to the vmPFC during combinatorial processing. This result suggests that a domain-general semantic combinatorial mechanism operates during basic linguistic composition, and that activity generated by its processing localizes to the vmPFC. In the second task, we recorded neural activity as subjects performed simple addition between two small numerals. Consistent with a wide array of recent results, we find no effects related to basic addition that coincide with our linguistic effects and instead find increased activity localized to the intraparietal sulcus. This result suggests that the scope of the previously identified linguistic effects is restricted to compositional operations and does not extend generally to all tasks that are merely similar in form.

Determining how language relates to cognition more generally is vital both for understanding how language works and how it came into being. Answers to questions regarding the origin of language in large part depend on the delineation between language specific and domain-general mechanisms (Hauser et al., 2002; Pinker and Jackendoff, 2005). An accurate determination of this line can help shed light on the magnitude and form of the evolutionary jump necessary to bring about linguistic ability (Marcus, 2006). Further, determining the relationship between language and cognition can help to calibrate the confidence with which findings from other domains and even species can be extended to language (Wise, 2003; McElree, 2006). If basic linguistic processes are utilized across many domains, this places restrictions on the form of the underlying mechanisms (i.e., it cannot be narrowly linguistic in nature). In the present experiment, we use magnetoencephalography (MEG) to assess the degree to which neural signatures previously shown to reflect basic linguistic composition, such as between an adjective and a noun, are also elicited by similar processing in non-linguistic domains.

While an abundance of evidence suggests that neural resources utilized during linguistic combinatorial processing are also active within non-linguistic domains, these studies have primarily investigated the processing of relatively complex linguistic structures that quite intuitively might recruit domain-general mechanisms such as executive control, attention, conflict resolution, and memory processes. For example, center embedding expressions, as in *the juice that the child enjoyed stained the rug*, are well known to reliably produce increased activity in the left inferior frontal cortex (Broca’s area) when compared to simpler constructions (Stromswold et al., 1996; Caplan et al., 2000; Grodzinsky and Santi, 2008). However, beyond basic linguistic composition, sentences of this type also require listeners to select between potential antecedents, modulate attention between locations in the sentence, and retrieve past words or phrases from memory. Non-linguistic tasks that tap into each of the mechanisms involved in comprehending such expressions – selection and cognitive control (Badre and Wagner, 2007), attention (Nelson et al., 2009), and memory (Fiebach et al., 2007) – have also been observed to generate increased activity in Broca’s area, along with a multitude of other tasks spanning a wide range of domains and abilities (Duncan and Owen, 2000; Thompson-Schill et al., 2005). Further, a direct comparison between the processing of syntactically ambiguous sentences and the resolution of stroop conflict identified coincident activity within this region (January et al., 2009). Other types of complex sentences, such as those that require the listener to resolve syntactic ambiguity in an unfamiliar manner, activate wide neuronal networks shared by non-linguistic processes, such as motor coordination, error monitoring, and response selection (Stowe et al., 2004). Within electrophysiological paradigms, ERP effects canonically elicited by various types of linguistic violations have been evoked within non-linguistic domains as well, such as music (Patel et al., 1998), pictures (Cohn et al., 2012), and using abstract geometric patterns (Besson and Macar, 1987). Thus, there is a large body of evidence indicating that language comprehension interacts with and relies upon domain-general operations at some level (though, see Fedorenko et al., 2011), however, it is presently unclear how deeply this interrelationship permeates linguistic processing. Specifically, it remains unknown to what extent basic linguistic combinatorial mechanisms that lie at the heart of language comprehension operate within non-linguistic domains as well.

In the present work, we use MEG to investigate the domain-generality of such operations, and we examine the extent to which simple combinatorial processing brought about by pictures and numbers evokes effects similar to those observed during basic linguistic composition. In a previous language paradigm (Bemis and Pylkkänen, 2011, 2012 and, see Figure 9 for a summary) we assessed linguistic combinatorial processing by identifying increases in neural activity evoked during the composition of an object-denoting noun with a color-denoting adjective (“red boat”) and compared this activity to that evoked by the same noun in a non-combinatorial context, such as when it was preceded by a non-pronounceable consonant string (“xhl boat”), a burst of auditory noise, or, critically, a different object-denoting noun, presented during a non-combinatorial task (“cup, boat”). Crucially, in this paradigm no judgments were required on the critical stimuli – subjects were only asked to comprehend naturally and then judge whether a following picture matched the preceding verbal description. We then only analyzed the activity generated during the critical nouns, which were identical across all conditions. During the composition of an adjective with a noun we found increased activity localized to the left anterior temporal lobe (lATL) at ∼200–250 ms and the ventro-medial prefrontal cortex (vmPFC) at ∼400 ms (Bemis and Pylkkänen, 2011), with a potential additional contribution from the left angular gyrus (lAG) at a similar later time (Bemis and Pylkkänen, 2012). All three of these regions have been implicated in combinatorial linguistic processing by a substantial number of studies (Bottini et al., 1994; Humphries et al., 2001; Vandenberghe et al., 2002; Mar, 2004; Lau et al., 2008; Fedorenko et al., 2011; Pallier et al., 2011), and thus these results provide a basis against which to judge the similarities between non-linguistic and linguistic combinatorial processing.

To assess the extent to which these effects generalize to non-linguistic domains, we replaced the word stimuli of these prior experiments with pictures and numbers. Pictures were chosen for their similarity to words in activating conceptual representations. Intuitively, both comprehending sentences and viewing visual scenes are capable of eliciting complex conceptual representations in the mind of the comprehender or perceiver, though little is known about the neural overlap in the two routes taken by these processes from perceptual input to conceptual realization. In the present study, we paralleled our linguistic task and investigated only minimal combination, as brought about through the viewing of a single colored shape. If the composition of a simple phrase such as “red boat” involves a semantic combinatory mechanism that is not dependent on linguistic input, then a picture of a red boat should engage the same mechanism, assuming a task that demands on the construction of a combined “shape + color” concept. In contrast, a picture of a red boat should not give rise to any specifically syntactic combinatory mechanisms, such as the construction of a noun phrase, as such mechanisms should be dependent on syntactic category information absent in pictures. Thus, any shared combinatory effects between words and pictures would support a semantic as opposed to a syntactic role for the implicated region.

As a second non-linguistic task, we chose a simple arithmetic task in which the “combinatorial” operation was the addition of two small numbers. While this task echoes aspects of the linguistic design, such as minimality and the functional transformation of two input elements to a single output, theoretical models of addition (Dehaene et al., 2003) do not generally include specific combinatorial mechanisms found in models of linguistic composition (Heim and Kratzer, 1998), such as the construction of syntactic phrases or the creation of new meanings. Thus, if this task elicited effects similar to those found for linguistic composition, it would suggest that the underlying mechanisms are of the most general combinatory nature, only loosely related to linguistic notions of syntactic and semantic composition. Thus, in sum our picture experiment aimed to assess whether combinatorial mechanisms involved in the comprehension of basic adjective-noun phrases are also involved in the construction of domain-general meaning, and the math experiment is designed to examine the extent to which these mechanisms may support very general combinatory mechanisms that convert multiple inputs into a single output regardless of their domain. This investigation diverges from prior studies into the domain-generality of linguistic composition in its simplicity: in each task we explicitly manipulate only a single step of composition.

## Experiment 1: Pictorial Combination

The purpose of the pictorial manipulation was to explore the extent to which basic combinatorial linguistic mechanisms are utilized for non-linguistic stimuli. While language is by far the most common method of constructing complex ideas (or meaning) out of simpler pieces, the processing of visual scenes (such as when viewing the external world) can also evoke similar mental representations (i.e., those that encode complex relationships between abstract concepts), and this cognitive process requires combination between individual conceptual elements as well. While there has been extensive investigation into the neural relationship between picture processing and lexical access during language production (Indefrey and Levelt, 2004), to date there has been little work that attempts to measure the construction of complex conceptual representations from pictorial stimuli. There has, however, been a large amount of work investigating perceptual combination, or feature binding, during visual object perception (Treisman and Gelade, 1980; Treisman, 1999; Quinlan, 2003), however, such processes are not the intended object of investigation for the current study. In general, this work has attempted to characterize the mechanisms by which perceptual features of a visual object are bound together into a single representational unit. In other words, when we perceive a picture of a red boat, we extract a percept of the color associated with the object (a particular shade of red) and a percept of the shape associated with the object (a particular type of boat). These percepts are thought to be initially extracted separately (Treisman and Gelade, 1980), and thus must later be established as belonging to the same object in the world. In general, feature binding refers to this establishment procedure. By contrast, when constructing the meaning of the linguistic expression *red boat*, we extract the concept of redness (not necessarily tied to a particular shade of red) and the concept of boatness (not necessarily tied to a particular type of boat), and then combine these separate concepts into an abstract representation of a single conceptual object. There are, of course, recent claims of synonymy between these two processes (Wu and Barsalou, 2009; Lynott and Connell, 2010), however, this equivalence has yet to be conclusively demonstrated empirically. With this distinction in mind, we have designed the present paradigm to maximize conceptual processing during picture viewing, as we believe it is this process that is most likely to occur during both language processing and combination evoked by pictorial stimuli. It may still be the case, however, that combinatorial effects observed during this latter type of processing reflect perceptual combination to some degree. To evaluate this possibility, in addition to the linguistic ROIs we have included a posterior region in the analysis as well that has been heavily tied to perceptual processing (the left mid fusiform gyrus, lMFG – Tarkiainen et al., 1999; Grill-Spector, 2003).

To date, there have been very few investigations into the extent that linguistic conceptual combinatorial mechanisms are utilized when processing pictorial stimuli. Perhaps the most relevant studies are those that have replicated the paradigmatic N400 effect using incongruent picture stimuli instead of sentence with incongruent endings (Nigam et al., 1992; West and Holcomb, 2002; Cohn et al., 2012). Unfortunately, it is difficult to draw a direct connection between this result and our previous linguistic findings as this paradigm differs from our own in several important respects. First, activity is measured during the comprehension of relatively complex meanings (i.e., descriptions of action sequences unfolding over time) and second, effects are associated with the processing of unexpected stimuli. In contrast, our linguistic paradigm was specifically designed to isolate minimal combinatorial processing in a normal context. Thus, in order to determine the extent to which our previous results might reflect domain-general mechanisms, we designed a parallel non-linguistic paradigm that sought to maintain these crucial qualities using pictorial stimuli.

**Figure 1:**
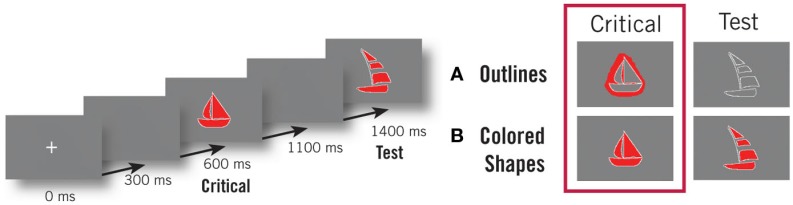
**Pictorial experimental design**. The trial structure (shown at left) was the same across both conditions. Subjects first saw a critical shape (either a colored picture or an outline on a colored background) and were asked to judge if a following test shape matched the initial stimulus. On Outline trials **(A)**, only the shape was required to match. On Colored Shape trials **(B)**, both the color and shape were required to match. Only activity evoked during the viewing of the critical stimuli was subject to analysis. Subjects saw an equal number of matching and non-matching trials in both conditions.

Specifically, we aimed to evoke semantic combination that was as alike as possible to the natural, simple intersective modification required by the prior linguistic manipulation but using non-linguistic pictorial stimuli. To this end (Figure 1), we replaced adjective-noun combinations with colored pictures depicting the color and shape denoted by the adjective and noun (e.g., “red boat” was replaced by a picture of a red boat) while single noun stimuli were replaced by simple outlines (e.g., “xtp boat” was replaced by an outline of a boat). Subjects were then asked to judge if a following test picture matched the preceding stimuli in both shape and color (for colored shape trials) or shape alone (for outline trials). Importantly, to avoid simple perceptual matching between the test and critical pictures, and so force subjects to perform conceptual combination, different tokens of each shape were used for the two sets of items. Thus items qualifying as matching were unambiguously of the same conceptually category (e.g., a boat), however they did not have the identical shape. Further, critical and test items were given different scalings and rotations to enhance the differences in form. Consequently, subjects could not simply perceptually compare the two pictures to each other. In order to assess the correspondence between the critical and task pictures in colored shape trials, subjects had to create a combined, conceptual representation of the color and shape percepts evoked by the critical picture beyond the lower-level binding of these percepts to a single object. It is this conceptual combinatorial processing that we expect to be functionally similar to that evoked by a linguistic adjective-noun combination. Though it should be noted that the identification of perceptual combinatorial effects within linguistic ROIs would be informative with respect to linguistic compositional mechanisms as well (cf. Wu and Barsalou, 2009). Conceptual combination was minimized during outline trials by requiring only the shape of the two pictures to match and by removing all color from the task pictures, thus eliminating the potential use of conceptual combination in the task. Thus, in sum, the present design attempted to use non-linguistic stimuli to evoke the creation of a complex conceptual object constructed by modifying an initial shape property with a color property; in other words, the same combinatorial semantic operation involved in the composition of an adjective and a noun. Additionally, we sought to evoke this combinatorial processing through as natural a context as possible while still maintaining the minimal simplicity of the previous design. We expect conceptual combinatorial processing to occur only during the processing of the colored shapes while outline stimuli should not evoke such processing. Consequently, if any of our previously identified combinatory effects reflect domain-general semantic composition, we would expect a similar effect to be elicited for the color-shape combinations in the present task as well.

There is both a theoretical and practical reason that combination was evoked from this subtle manipulation and not through the sequentially presentation of two pictures (i.e., a color spot followed by a shape outline). First, we wanted to maintain the naturalness of the linguistic experiment to the greatest degree possible, and, in general, people view complex visual objects all at once and not as two sequential presentations. Second, pilot testing determined that subjects were much more likely to use an explicit verbalization strategy when presented with two sequential stimuli compared to a single picture. As the present study is designed to investigate conceptual combination in the pictorial domain, this finding played a substantial role in deciding to present a single unified picture and not a sequentially broken version. Additionally, at no point were subjects told to expect linguistic stimuli or was any reference made to the specific names of any object during the instructions.

### Materials and methods

#### Participants

Twenty-four non-colorblind, fluent English speakers participated in the study (16 women). All had normal or corrected-to-normal vision. Their average age was 23.1 years (18–42 range). The protocol was approved by the Institutional Review Board, and subjects provided written consent before beginning the experiment.

#### Stimuli

Subjects were presented with two trial types, Colored Shape and Outline, each of which contained an initial fixation, a critical shape, and a test shape (Figure 1). In Colored Shape trials, a subset of the test shapes from our previous linguistic experiment served as the critical stimuli (the bags, bells, boats, bows, cars, cups, houses, keys, lamps, leaves, locks, notes, planes, shoes, and trees). These pictures were manually created canonical depictions of the shapes denoted by each noun and each was filled with one of six colors (red, blue, pink, black, green, brown). Additionally, three versions of each picture were created by applying a random scaling factor between 105% and 115% and a random rotation of 0°–360° to the original Figure. Critical pictures in the Outline trials were created from these stimuli by transferring the color from the middle of the shape outline to an area around its border using Photoshop. Thus, the total number of colored pixels remained equal between the two sets of stimuli. In total, two different tokens of each shape type were used, one for the test shapes and one for the critical stimuli. No token was used in both sets and tokens were used in the same set in each experiment. In the Colored Shape trials, test images were filled with color, while in Outline trials tests were presented without color. At the beginning of each trial, a small cross appeared and was centered in the middle of the screen.

During the task, subjects viewed 240 trials, 120 of each trial type, and both trial types were mixed together. Both conditions contained an equal number of trials for which the test shape matched or did not match the preceding shape. For Colored Shape trials, the non-matching trials were divided equally among those that did not match the color and those that did not match the shape. Additionally, in these trials all test shapes matched at least one feature of the preceding shape. During each condition, each of the 15 possible description shapes was used eight times (each time randomly paired with one of the six possible colors), four times in matching trials and four times in non-matching trials. Trials and stimulus lists were randomized and constructed separately for each subject.

#### Procedure

Before the experiment, subjects practiced the task outside of the MEG room. Prior to recording, subjects’ head shapes were digitized using a Polhemus Fastrak 3D digitizer (Polhemus, VT, USA). The digitized head shape was then used to constrain source localization during analysis by coregistering five coils located around the face with respect to the MEG sensors. During the experiment, subjects lay in a dimly lit, magnetically shielded room. All stimuli were presented using PsyScope X and were projected onto a screen ∼50 cm from the subject’s eye. Shapes subtended between 6° and 10°. Each trial began with the presentation of a fixation cross for 300 ms, a blank screen for 300 ms, and either a colored shape or an outline on a colored background for 500 ms. This picture was then followed by a 300-ms blank screen and the test picture, which remained onscreen until the subject’s response. Inter-trial intervals followed a normal distribution with a mean of 400 ms and a standard deviation of 100 ms.

Neuromagnetic fields were recorded continuously with a whole-head, 157-channel axial gradiometer array (Kanazawa Institute of Technology, Nonoichi, Japan) at a sampling rate of 1000 Hz in a band between 0 and 200 Hz, with a notch filter at 60 Hz. The entire recording session lasted ∼25 min.

#### Data acquisition

Magnetoencephalography data evoked by the initial shape, from 100 ms before to 600 ms after its onset, were segmented out for each subject for each condition. These data were cleaned of potential artifacts by rejecting trials for which the subject answered either incorrectly or too slowly (defined as over 2.5 s after the appearance of the colored shape) or for which the maximum amplitude exceeded a threshold that varied between 2500 and 3500 fT depending on the amplitude range of each subject. Overall 9.1% (3.5% std.) of trials were excluded due to behavioral error or artifacts. Remaining data were then averaged for each subject for each condition and band-pass filtered between 1 and 40 Hz. For inclusion in further analysis, we required that subjects show a qualitatively canonical profile of evoked responses during the processing of the critical items. This profile was defined as the appearance of robust and prominent initial sensory responses. In the visual modality, we required the presence of either the M100 or M170 field pattern (Tarkiainen et al., 1999; Pylkkänen and Marantz, 2003) in the time window of 100–200 ms following the onset of the critical stimuli. In order to assess this criterion, preliminary grand average waveforms were constructed for each subject by averaging over both conditions. Five subjects failed to meet this requirement and were excluded from further analysis.

#### Data analysis

In order to maximally parallel our previous linguistic paradigm, the analysis procedure in the present experiment was identical to that of Bemis and Pylkkänen (2011), which contains a more detailed description of the procedure and statistical tests used to analyze the data.

As in that study, distributed minimum norm source estimates served as our primary dependent measure in the present analysis. These were created for each condition average for each subject using BESA 5.1 (MEGIS Software GmbH). Each estimated resulted in 713 non-directional sources spread across a smoothed cortex for which electrical activity estimates could be compared between conditions at each time point. Our main analysis then attempted to identify significant effects in activity that localized to the regions of interest (ROIs) previously associated with linguistic combination – the lATL, vmPFC, and lAG. While the use of different subjects across experiments precludes a direct comparison of localized activity, we attempted to minimize differences by maintaining the exact boundaries of the ROIs as used in Bemis and Pylkkänen (2011, 2012). These boundaries were initially based upon hemodynamic results demonstrating increased activity in the lATL and lAG during the comprehension of sentences compared to word lists (Mazoyer et al., 1993; Friederici et al., 2000; Vandenberghe et al., 2002) and MEG results associating increased activity in the vmPFC with the resolution of semantic mismatches (Pylkkänen and McElree, 2007; Brennan and Pylkkänen, 2008, 2010). Additionally, we investigated potential differences in perceptual processing that might arise from the different types of visual stimuli used in the Colored Shape and Outline trials by drawing a broad ROI centered roughly around the lMFG. This region has been identified as integral to the perceptual processing of many types of complex visual stimuli (Tarkiainen et al., 1999; Grill-Spector, 2003), and so effects due to perceptual processing are likely to manifest in this ROI. Following our earlier study, the boundaries for this ROI were based on previous functional results and encompassed a rather broad cortical region in order to capture as much relevant activity as possible. Researchers wishing to reproduce this region can contact the corresponding author for exact dimensions regarding this ROI and all other ROIs used throughout the study. We also performed a supplemental full-brain analysis to identify differences in activity beyond these predefined ROIs.

Differences in activity localized to each ROI were identified using a non-parametric, cluster-based permutation test (Maris and Oostenveld, 2007) with the same parameters as in Bemis and Pylkkänen (2011, 2012). The primary advantage of this test is that it can determine time windows of significantly different activity across the entire epoch while controlling for multiple comparisons. Thus, we did not need to specify precise temporal ROIs *a priori*. Briefly, this test works by first calculating a single test statistic for the observed data and then comparing this value to a distribution bootstrapped from randomly permuting the original data. In this analysis, the test statistic was calculated by first determining contiguous time points for which the difference between conditions at each time point reached a certain statistical threshold (*p* = 0.30 by a two-tailed, paired samples *t*-test) and then summing the *t*-statistics from each point-wise comparison within the cluster. Permutations of the data were then created by randomly assigning the condition labels within each subject. The same test statistic was then calculated for each of these permutations, and the *p*-value of the observed test statistic was set equal to the proportion of permuted data sets that produced a larger test statistic. All *p*-values reported in the results were derived from distributions of 10,000 permutations of the original data.

The primary purpose of the full-brain analysis was to supplement the ROI findings and confirm that effects observed within an ROI occurred primarily within the predefined boundary. Additionally, we sought to obtain a broad overview of any combinatory effects that occurred outside of these regions. Thus, we compared activity estimates at each source-time point in the epoch using a two-tailed, paired samples *t*-test and then applied a liberal set of statistical, spatial, temporal, and amplitude filters to the results to eliminate spurious activity differences. The cutoffs for these filters were set at *p* = 0.05, five contiguous sources, five contiguous time points, and at least a 1 nAm difference between the activity in each condition, averaged across all subjects. In the results and figures below, we discuss only effects attributable to an increase in activity during the combinatorial Colored Shapes condition compared to the Outline condition.

### Results

#### Behavioral results

**Figure 2:**
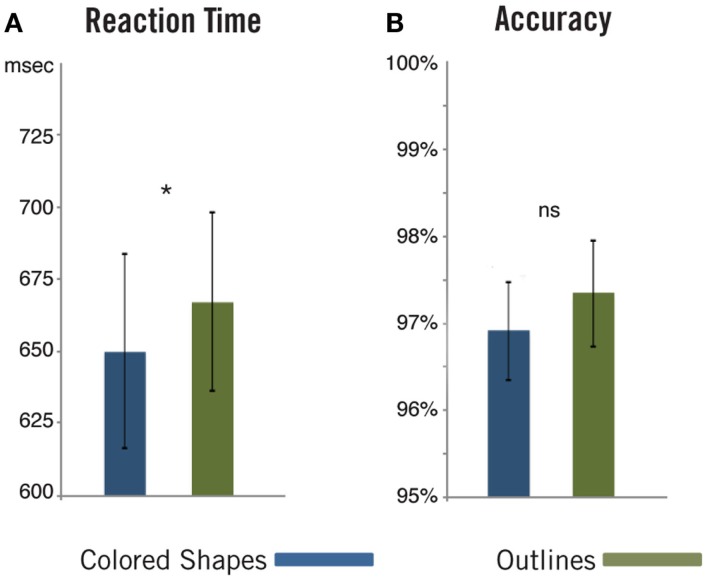
**Pictorial behavioral results**. As in our previous linguistic paradigm, reaction times **(A)** showed that subjects were significantly faster to judge matching against a combined property (Colored Shapes) than against a singular property (Outlines). We found no difference in accuracy **(B)** between the two conditions. ns, Non-significant; *p < 0.05.

Reaction time and accuracy data (Figure 2) were submitted to paired samples *t*-tests. We found no significant difference in accuracy between responses in Colored Shape trials (*M* = 96.9%, SD = 2.5%) and Outline trials (*M* = 97.3%, SD = 2.7%), *t*(18) = 0.98, *p* = 0.34 (two-tailed). We did, however, observe significantly faster responses in Colored Shape trials (*M* = 649 ms, SD = 147 ms) compared to Outline trials (*M* = 666 ms, SD = 135 ms), *t*(18) = 2.55, *p* = 0.02 (two-tailed). Past studies have demonstrated faster responses when subjects judge pictures against composed, complex linguistic descriptions as opposed to against simple, one-word descriptions (Potter and Faulconer, 1979; Bemis and Pylkkänen, 2011, 2012). Thus, the present result would be expected if linguistic composition caused this prior facilitation, and subjects performed a similar combinatorial operation in the current Colored Shape trials, as intended. Alternatively, responses on the Outline trials may have been slower due to a mismatch between the presence and absence of color information on the critical and test shapes respectively. This possibility is explored in more depth within the discussion below.

#### ROI results

**Figure 3:**
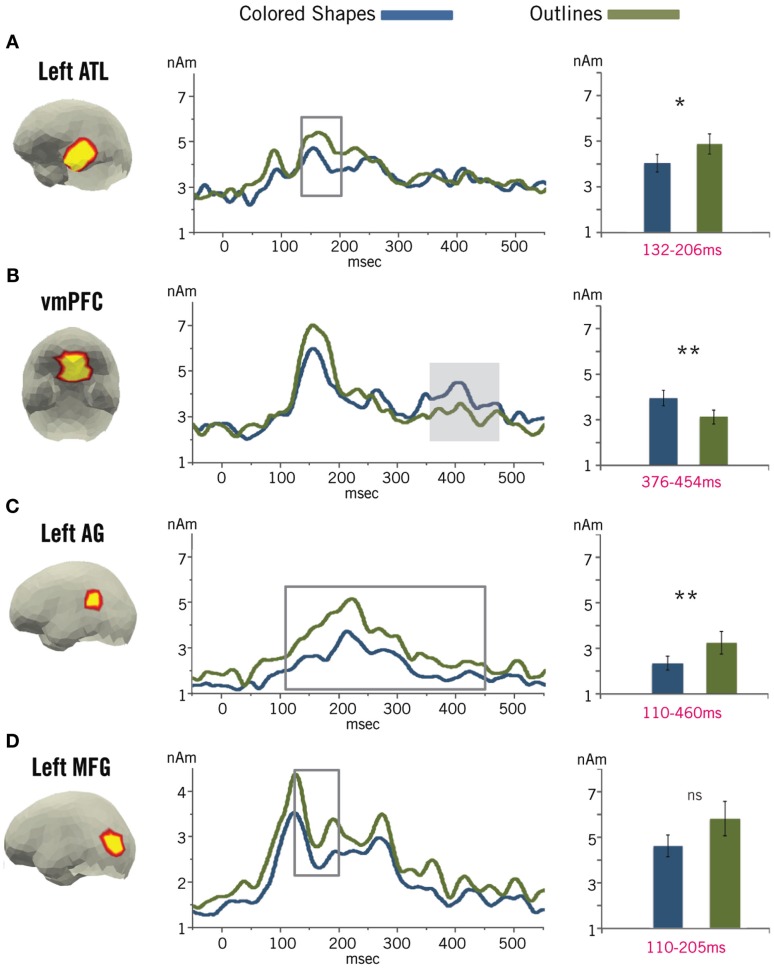
**Pictorial ROI results**. Localized activity is shown for the three ROIs for which we observed significant combinatorial activity during basic linguistic composition – the left ATL **(A)**, vmPFC **(B)**, and left AG **(C)** – and one ROI intended to capture activity related to perceptual processing of the stimuli – the left MFG **(D)**. The shaded region denotes a significant cluster of increased activity in the vmPFC during the Colored Shape condition, as identified by a permutation test (Maris and Oostenveld, 2007). The boxed regions denote clusters of increased activity during the Outline condition for the other ROIs. Only the cluster in the left AG was significant, while those in the left ATL and left MFG were marginal. ns, Non-significant; *p < 0.05; **p < 0.01.

Figure 3 displays the time course of activity localized to the four ROIs during the processing of the critical stimuli. Only activity localized to the vmPFC ROI exhibited the profile expected for a combinatorial mechanism. In this ROI, we identified a significant cluster (376–454 ms) of increased activity in the Colored Shape condition (*M* = 3.75 nAm, SD = 1.41 nAm) compared to the Outline condition (*M* = 2.96 nAm, SD = 1.25 nAm), *p* = 0.0475 (10,000 permutations). No significant clusters of increased activity in the Outline trials were identified at any point in the epoch for activity localized to the vmPFC (all clusters, *p* > 0.10).

On the other hand, we did identify clusters of increased activity during the Outline trials compared to the Colored Shape trials localized to the other three ROIs. This effect was only significant in the lAG ROI, where we identified a significant cluster from 110 to 460 ms of increased activity in the Outline condition (*M* = 3.09 nAm, SD = 2.01 nAm) compared to the Colored Shape condition (*M* = 2.24 nAm, SD = 1.26 nAm), *p* = 0.0003 (10,000 permutations). We identified marginally significant clusters in the lATL (132–206 ms, *p* = 0.0957, 10,000 permutations) and lMFG (110–205 ms, *p* = 0.0969, 10,000 permutations) with increased activity in the Outline trials (lATL: *M* = 4.62 nAm, SD = 1.82 nAm; lMFG: *M* = 5.50 nAm, SD = 3.07 nAm) compared to the Colored Shape trials (lATL: *M* = 3.83 nAm, SD = 1.59 nAm; lMFG: *M* = 4.37 nAm, SD = 1.93 nAm). We identified no significant clusters of increased activity in the Colored Shape trials compared to the Outline trials for the lATL, lAG, or lMFG ROIs (all clusters, *p* > 0.10).

#### Full-brain results

**Figure 4:**
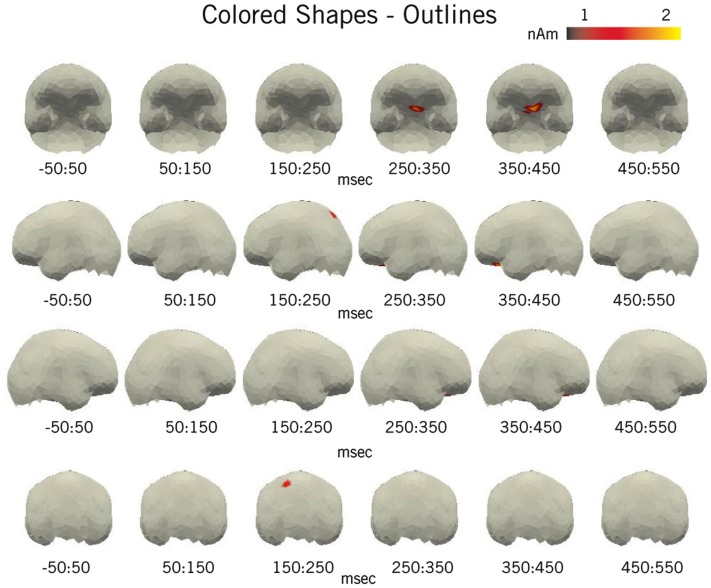
**Pictorial full-brain results**. Plotted regions denote the difference in average amplitude between the Colored Shape and Outline conditions for all space-time regions in which Colored Shape activity was reliably greater than Outline activity (by at least 1 nAm and *p* < 0.05, paired samples *t*-test) for at least 5 ms over five spatial neighbors. For clarity, non-cortical sources have been removed. Results conform to our ROI analysis and show a clear vmPFC effect at ∼400 ms. An additional increase in activity generated during the Colored Shape condition can be seen in the superior parietal cortex at ∼200 ms.

Our full-brain analysis (Figure 4) confirms the combinatorial results from the ROI analysis, as a clear increase in activity during Colored Shape processing can be seen localized to the vmPFC from 300 to 400 ms. Further, this effect is located entirely within the predefined vmPFC ROI. Also, as in the ROI analysis, there are no observable activity increases in the full-brain analysis localized to the LATL, AG, or lMFG.

Outside of the predefined ROIs, the only visible increase in activity occurred relatively early (∼200 ms) and was localized in the left superior parietal cortex. This region has long been implicated in the control of spatial attention, especially during the processing of visual stimuli that require attention to multiple features (Corbetta et al., 1995). Thus, this activity may reflect an increase in attention during the processing of the colored shapes, potentially driven by the need to initially bind the color and shape percepts into a complex visual representation prior to conceptual combination.

### Discussion

The present experiment was designed to investigate whether the neural mechanisms that support basic linguistic composition operate during non-linguistically driven combination as well. Thus, our pictorial paradigm paralleled our previous linguistic design (Bemis and Pylkkänen, 2011), and we recorded neural activity as subjects viewed a description of a colored shape prior to judging whether a following test shape matched the preceding description. In this case, however, the descriptions were pictorial rather than linguistic, and subjects had to determine whether the two stimuli were of the same conceptual class (i.e., the same shape type and color). Importantly, subjects could not simply perceptually match the two shapes to each other as we used different tokens of each type for the description and test pictures. Thus subjects were compelled to construct a more abstract complex representation, similar to that elicited by the composition of an adjective and a noun. Combinatorial activity was isolated by comparing the processing during these colored shapes to that evoked by a shape outline on a colored background. To minimize combinatorial processing in this condition, subjects judged only if a following outline was of the same shape type. We identified increased activity during the combinatorial condition localized to one of the regions also implicated in basic linguistic composition, the vmPFC ROI. This result suggests that the combinatorial mechanism subserved by this region during basic linguistic combinatorial processing operates outside the linguistic domain and is involved in conceptual combination more generally.

While our previous study is not alone in linking the vmPFC to combinatorial language processing (Mar, 2004), this region has canonically been associated with a variety of non-linguistic tasks. Increased activity in this region has been observed during theory of mind tasks (Sabbagh et al., 2004), emotional processing (Northoff et al., 2000), error monitoring (Bayless et al., 2006), and the calculation of reward and punishments (O’Doherty et al., 2001). Thus, our finding that a non-linguistic task drives activity in this region is broadly consistent with a large body of evidence implicating this region in non-linguistic processing. Our results do however more closely align at least one of the functions subserved by this region with basic linguistic processing. Specifically, the results of Bemis and Pylkkänen (2011) combined with the present results suggest that the vmPFC supports a combinatorial mechanism that operates during both basic linguistic composition and conceptual combination evoked by simple colored pictures. As the latter processing does not involve any syntactic composition, these results suggest that the vmPFC supports the composition of semantic representations during both linguistic and non-linguistic processing; a conclusion consistent with past work implicating this region in the resolution of grammatically correct but semantically mismatched expressions (Pylkkänen and McElree, 2007; Brennan and Pylkkänen, 2008).

It should be noted, of course, that these results do not imply the vmPFC houses the sole neural mechanism responsible for combining meaning. Even within language alone, the composition of two concepts requires a large and diverse set of cognitive operations (Murphy, 1988; Kamp and Partee, 1995), and the general ability to combine concepts surely involves the interactive effort of multiple neural regions. Therefore, it is unlikely that damage to any one single neural region will entirely incapacitate this ability. However, there remains a tension between the interpretation of the present results and the neurophysiological literate that primarily links vmPFC damage to deficits in impulse control (Mesulam, 2002), social interactions (Bechara et al., 1994), and cognitive control (Fellows and Farah, 2003). In combination with the neuroimaging results cited above, these findings suggest that the vmPFC plays an important role in complex, goal-oriented behaviors as well basic combinatorial operations. Taken as a whole, the vmPFC encompasses a rather large cortical region, which is both cytoarchitecturally heterogeneous and widely interconnected to a variety of both subcortical and cortical regions (Hof et al., 1995; Ongür and Price, 2000). Thus, it is quite likely that distinct neural subpopulations within this region participate in several different cognitive operations. Given the widely acknowledged propensity of the brain to reorganize following damage (Chen et al., 2002; Saur et al., 2006), the relative lack of deficits observed for basic combinatorial processing compared to goal-directed behavior following damage to the vmPFC may suggest a less centralized and more redundant neural complex supporting the former compared to the latter. Further work is clearly needed to disentangle the interaction of functional heterogeneity and neural reorganization following damage within this region. In particular, we believe it would be highly relevant to our interpretation to observe changes in the activity profile for both the present manipulation and our previous linguistic paradigm for patients with damage to the vmPFC, particularly across different time periods following trauma, in order to chart reorganization as a function of time (cf. Saur et al., 2006).

Despite this outstanding puzzle, our results suggest that non-linguistic conceptual combination recruits neural mechanisms that are active during basic linguistic composition as well. One consideration in drawing this conclusion is that combinatorial effects observed in the present contrast might reflect perceptual as opposed to conceptual combination. This possibility cannot be ruled out entirely from the present design, and we did, in fact, identify increased activity in the combinatorial condition localized to the superior parietal cortex, which has been linked to directing spatial attention and visual feature binding (Corbetta et al., 1995). However, this effect occurred earlier than that observed in the vmPFC, falling within the time frame canonically associated with visual processing (Tarkiainen et al., 1999). Contrastingly, neither the timing nor the location of the observed combinatorial effect – in the vmPFC at ∼400 ms – match the spatio-temporal profile of canonical visual effects, which occur earlier and in more posterior regions (Tarkiainen et al., 1999; Grill-Spector, 2003). Such an effect was instead observed in the Outline condition for activity localized to the lMFG from ∼100 to 200 ms. We also observed increased activity in the lAG and lATL ROIs during this condition, as well as slower reaction times at the test shape. One possibility is that this entire constellation of effects is related to the need to suppress color information, present within the critical shapes, during the decision at the test shapes, which do not contain color. Under this hypothesis, increased activity observed in the lMFG might reflect increased perceptual processing required to separate figure from ground (Schira et al., 2004), and activity localized to the lATL and lAG, both regions previously linked to single word lexical processing as well as combinatorial linguistic tasks (Horwitz et al., 1998; Mummery et al., 1999; Binder et al., 2000; Fujimaki et al., 2009), including covert object naming (Ellis et al., 2006), may reflect the enlistment of a verbal strategy in maintaining a separation between the color and shape information (cf. Sperling, 1963). While such extra processing during the Outline trials was not intended, it does suggest that Outlines, and not Colored Shapes, evoked more activity related to perceptual processing, and therefore the observed effect in the vmPFC is more likely to reflect conceptual as opposed to perceptual combinatorial processes.

There is the danger, of course, that our paradigm erred too far toward paralleling our previous linguistic experiment and simply evoked linguistic combination as before, through the covert use of verbalization to solve the task. Though informal interviews following the recording session suggested that subjects were not aware of such verbalization, introspective reports can hardly be taken as definitive evidence against this possibility (Dodge, 1912; Schwitzgebel, 2008). Likewise, while the absence of an lATL effect suggests a dissociation in processing compared to the previous experiment, this null result is at best suggestive and may reflect either a loss of power or more variability in the data. Thus, the present results do not entirely rule out the possibility that subjects relied upon a verbal strategy to complete the combinatorial pictorial task. It must be noted, however, that the question of whether or not subjects used a “verbal” or “non-verbal” strategy during the combinatorial task must be posed with care as it is, in fact, precisely the fusion of these two notions that we are interested in investigating, or, more exactly, the point at which they fuse together. Based on our results, we are suggesting that this point occurs during the construction of complex semantic representations. Specifically, we are suggesting that during non-linguistic conceptual combination, as brought about in the present task, a pictorial description activates two conceptual representations, such as those that encode *red* and *boat*, and that their subsequent combination makes use of a neural mechanism also operational during the linguistic composition of an adjective and a noun. This latter process, in highly simplified terms, involves the recognition of a linguistic item (such as a visually presented word), followed by the retrieval of information from the lexicon, followed by the activation of conceptual representations that are again bound together, in conjunction with syntactic structures, through the use of the same combinatorial mechanism. Thus, at a certain point, in this case conceptual combination, the “verbal” and “non-verbal” strategies become synonymous.

Two caveats to this suggested interpretation should be mentioned. First, though our results suggest a shared domain-general combinatorial process between linguistic and non-linguistic processing, the present data remains equivocal on the exact nature of the shared mechanism. While we are suggesting that the junction between linguistic and non-linguistic processing streams occurs at semantic composition, the point of contact may in fact occur earlier. For instance, the pictorial representations in the combinatorial task may activate lexical precursors to semantic composition, thus sharing the linguistic combinatorial route from this point forward. Further work is needed in order to more precisely isolate the moment of contact. Second, while the present results suggest the engagement of a linguistic combinatorial mechanism, they do not speak directly to whether or not this engagement is essential for completing the non-linguistic task. In other words, though our results indicate that a non-linguistic combinatorial task initiates the involvement of a linguistic combinatorial mechanism, the present paradigm cannot determine if this involvement is *necessary* for the completion of the task and thus whether the initiated process constitutes a truly domain-general mechanism, i.e., one that is indispensible for performing both the non-linguistic and linguistic task. One popular approach for assessing the necessity of linguistic processing is through the use of concurrent verbal shadowing (e.g., Hermer-Vazquez et al., 1999). Unfortunately, at the present time not only is it unclear how neural activity generated by shadowing can adequately be differentiated from that of the main task but, again, we are interested in identifying a point of contact between linguistic and non-linguistic processing. Thus, the introduction of extra, explicitly linguistic processing, even if intended to be entirely non-combinatorial, will only complicate this determination.

Aside from the central question of shared combinatorial processing, there are also questions, which plague many psychological experiments, about the relationship between the processing evoked during the experimental task and that which occurs “in the wild.” For example, it is unclear if conceptual combination, of the type we intended to study, occurs whenever a visual object is attended to or if it is task-dependent. Further, it remains to be seen if the effects observed in the present paradigm persist during the processing of more complex visual scenes in more natural circumstances. Efforts in this direction are currently ongoing in both the linguistic (Brennan et al., 2012) and pictorial domain (Hasson et al., 2010), though a direct comparison between the two has yet to be performed. Thus, the results of the present experiment suggest that a common combinatorial neural mechanism lies at the heart of both basic linguistic and non-linguistic combination. Much work, however, remains in elucidating the precise nature of this mechanism and the extent to which these two processing streams coincide.

## Experiment 2: Mathematical Combination

In contrast to the picture experiment, which was designed to test a rather plausible hypothesis about shared semantic composition between language and pictures, our math experiment was designed to constrain the functional hypothesis space open to our previous findings, namely that they reflect highly general combinatory mechanisms engaged every time any computation needs to be performed on two symbols. Specifically, we sought to construct a paradigm similar in form to the previous linguistic task, but sufficiently different in function so as not to evoke the same combinatorial mechanisms. In other words, we desired to measure activity generated by an operation that converts two simple inputs into a single output, but that does not plausibly require the same compositional operations involved in basic linguistic combinatorics, such as syntactic or semantic combination. A negative result from such a task would support the hypothesis that the effects observed previously during linguistic composition reflect these more specific combinatorial mechanisms and are not always elicited by tasks that are merely similar in structure.

To this end, we chose a manipulation involving basic mathematical processing. Despite tantalizing similarities between the structure of language and mathematics, there is a growing body of evidence that suggests these two types of processing employ largely distinct mechanisms, especially in terms of combinatorial mechanisms. The superficial similarities between the two, however, are quite noticeable and have driven extensive investigation into the relationship between these two modalities. Like linguistic expressions, mathematical expressions exhibit hierarchical structural relations between basic elements, controlled by well-formedness rules (Friedrich and Friederici, 2009). Indeed, the most prominent mathematical processing model (Dehaene et al., 2003) posits a non-negligible overlap between linguistic and numerical processing. In this “triple-code” model, one of the three hypothesized codes underlying mathematical processing is an explicitly verbal representation of number. In support of this proposal, Dehaene et al. (2003) point out that certain types of mathematical deficits are often accompanied by aphasia (Dehaene and Cohen, 1997; Cohen et al., 2000). Additionally, the angular gyrus, often implicated in linguistic processing (Horwitz et al., 1998; Binder et al., 2000) including our own composition work (Bemis and Pylkkänen, 2012), shows increased activity during many types of mathematical processing (Dehaene et al., 1999; Fulbright et al., 2000). Finally, behavioral studies indicate that concurrent language tasks interfere with basic mathematical operations (Spelke and Tsivkin, 2001; Lee and Kang, 2002). Crucially however, the role of language in these mathematical tasks is not assumed to be combinatorial in nature. Instead, it is posited to reflect the retrieval of rote learned declarative facts through verbal associations (Dehaene et al., 2003). Linguistically-related effects have been observed primarily in mathematical tasks for which the answer has been learned and can be recalled without resorting to calculations involving magnitude (e.g., multiplication with small numbers). Such effects are absent or at least much reduced during similar types of operations for which the answer is not assumed to have been learned, such as simple subtraction problems (Chochon et al., 1999; Lee, 2000). Further, increased activity in traditionally linguistic cortical regions, such as the angular gyrus, has been shown to track the extent to which mathematical answers have been learned, showing more activity for exact compared to approximate problems (Dehaene et al., 1999) and smaller compared to larger sums (Stanescu-Cosson et al., 2000). Thus, despite the potentially integral role that linguistic representations play in certain types of mathematical processing, their hypothesized function is entirely distinct from combinatorial processing as employed during the comprehension of linguistic phrases.

This hypothesis has been put to the test on several recent occasions by attempting to produce canonical linguistic combinatorial effects using complex mathematical tasks, leveraging the potential hierarchical structural similarities between the two domains. To date, these attempts have failed to find effects during the processing of “combinatorial” mathematical expressions. Martín-Loeches et al. (2006) constructed “syntactic” violations parallel in structure to linguistic violation paradigms. Following Dehaene and Cohen (1995), they roughly equated mathematical operators (e.g., “+”) with function words and found that subsequent violations in which these operators appeared in syntactically incorrect locations failed to produce the anterior negativities that similar word-category violations do (Friederici et al., 1993), instead resulting in posterior parietal negativities. Similar results were obtained when examining ERP responses to mathematical parentheses, hypothesized to introduce “relative clause” like expectations (i.e., embedded combinatorial processing). An fMRI study of hierarchical mathematical expressions, compared to structurally flat ones, also failed to replicate effects found in parallel linguistic manipulations (Friedrich and Friederici, 2009). Finally, Varley et al. (2005) present patient data for which linguistic comprehension is severely impaired, while mathematical computation is preserved. These patients are able to correctly answer mathematical problems that depended on order (60–90 vs. 90–60) while not being able to distinguish parallel linguistic expressions (“John hit Mary” vs. “Mary hit John”) and can solve problems involving embedding [36/(3 × 2)] while not being able to comprehend embedded linguistic expressions (“The reporter that attacked the senator admitted the error”). Thus, to date, despite concerted efforts to show otherwise, there is no evidence that mathematical processing employs combinatorial mechanisms similar to those used in linguistic comprehension. It appears as though, despite superficial similarities in structural complexity, different processing mechanisms underlie the comprehension and parsing of complex mathematical and linguistic expressions.

**Figure 5:**
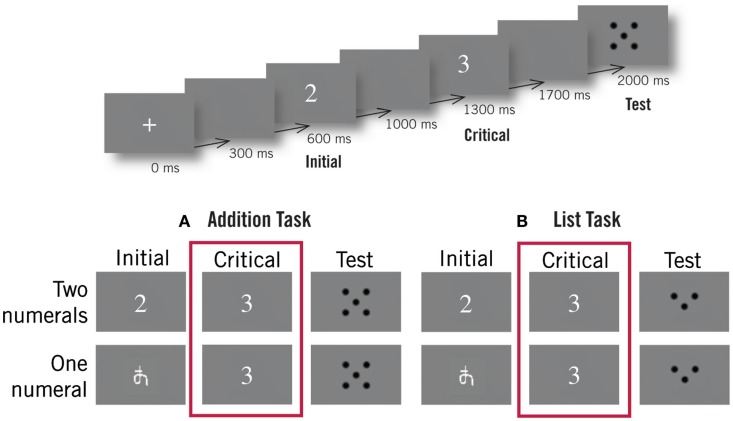
**Mathematical experimental design**. The trial structure (shown at top) was the same across all conditions. Subjects saw an initial stimulus (either a numeral, 1–5, or a non-sense symbol), a critical numeral (a numeral 1–5), and a test picture (a small set of dots). In the addition task **(A)**, they were asked to judge if a following set of dots was equal to the sum of all preceding numbers. In the list task **(B)**, they were asked to judge if the following set of dots matched any of the numbers. Only activity evoked during the viewing of the critical numerals was subject to analysis. Subjects saw an equal number of matching and non-matching trials in all conditions.

Thus, in order to explore the bounds of the effects observed in our combinatorial linguistic manipulation, we designed a parallel mathematical task that required the simple addition of two small numbers (Figure 5). Though similar in structure to basic linguistic combination, this task should be functionally distinct, at least in terms of combinatorial processing. To maximize the structural parallelism, the mathematical design replicated the linguistic design in full, which included not only a comparison between one and two-word trials (*red boat* vs. *xtp boat*) in a task invoking composition, but also a control “list task” that served as a lexical-semantic control. In this control task subjects read lists of one or two nouns (*xtp boat* vs. *cup*, *boat*) and judged whether a subsequent picture matched any previously presented noun. Mirroring this structure, our math design was split into two complementary tasks, each of which contained two and one-element trials. In each task, subjects were shown either two sequential, small (1–5) Arabic numerals or a visually matched meaningless symbol followed by a single digit. In the critical addition task, subjects were asked to judge whether or not a following set of dots was equal to the sum of the preceding numbers. In the control list task, subjects were asked to judge if the set of dots was equal to any of the preceding numbers. As can been seen, these two tasks follow the general form of the two linguistic tasks, and in both cases the critical trials require subjects to perform an operation requiring two operands (either an adjective and a noun or two-numerals) that were presented one after the other. Further, both the linguistic and mathematical operations are simple and basic, with no judgment required at the presentation of the critical stimuli. Therefore, if the effects observed during the linguistic paradigm reflect general mechanisms that operate during any process that maps two input elements onto a single output, then we would expect to see similar effects in the present paradigm. For example, previous effects may reflect either the retrieval or encoding of a single object in memory with multiple elements or cues. During combination, various levels of representation of *red* and *boat* may be associated with the resulting complex representation of a red boat just as representations of *2* and *3* may be associated in memory with their resulting sum. No such single object is as easily associable with the separate elements in the list conditions. Further, in the addition condition the sum of the two-numerals may in part be retrieved from memory through the mutual use of both addends, *2* and *3*, as cues (Dehaene et al., 2003) just as information related to a composed complex meaning may be retrieved from memory during language comprehension through the use of both linguistic elements. Again, no corresponding memory operation would be expected during processing in the list controls. On the other hand, if our previously observed linguistic effects reflect more specific combinatorial mechanisms, such as those required to construct complex syntactic or semantic representations, then we would not expect to observe the same effects in the present paradigm.

If this were to be our finding, then it would be desirable to obtain a positive result as well to demonstrate that the present paradigm does not lack the power to find effects related to basic addition. It is somewhat unclear, however, exactly what effects we should expect to see during simple addition. Within the triple-code model, basic addition as employed in our design is hypothesized to be solved either through the recall of memorized verbal facts or through calculation using the magnitude of the presented numbers (Dehaene et al., 2003). A wide range of data, however, indicates that mathematical calculation more generally produces increased activity in the intraparietal sulcus (IPS) with strong regularity (Chochon et al., 1999; Pesenti et al., 2000; Dehaene et al., 2004; Roitman et al., 2012). Therefore, as a sanity check for our task and to avoid a null result as the sole prediction from this manipulation, we included the IPS as a region of interest during the analysis of the mathematical data.

As a final note, it should be emphasized that while our design has the ability to assess whether or not previous effects observed in the lATL, vmPFC, and lAG during basic linguistic composition reflect the general form of our previous task (as opposed to combinatorial mechanisms) it does not, of course, have the ability to assess the domain-generality of all linguistic combinatorial mechanisms in relation to mathematics. While past evidence suggests that basic linguistic combinatorial mechanisms are not likely to be involved in basic addition, it may well be the case that shared mechanism exist at a more complex level of operation. Thus, a negative result with respect to the specific linguistic ROIs analyzed in the present experiment cannot be taken to imply a complete functional separation between linguistic and mathematical operations.

### Materials and methods

#### Participants

Twenty native English speakers participated in the study (12 women). All had normal or corrected-to-normal vision. Their average age was 25.6 years (18–43 range). The protocol was approved by the Institutional Review Board, and subjects provided written consent before beginning the experiment.

#### Stimuli

Each trial contained a small fixation cross, an initial numeral or symbol, a critical numeral, and a test set of dots (Figure 5). Numerals were Arabic numbers from 1 to 5 created in non-proportional Courier font. One-numeral trials were constructed from two-numeral trials by replacing the initial numeral with a matched symbol stimulus, manually created in Powerpoint and designed to not carry any obvious association to Arabic numerals (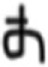, 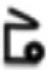, 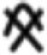, 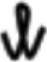, 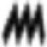). Numerals and symbols were sized to fill the same visual area and were matched in terms of total pixels on a 250 × 250 picture file [*p* = 0.63; *t*(4) = 0.52; two-tailed, paired samples *t*-test; numerals: *M* = 90.4, SD = 16.1; symbols: *M* = 91.0, SD = 14.8]. Sets of dots were constructed using small black circles. Each set of dots was arranged as on a die, except for the set of three, which formed a triangle instead of a line. Sets greater than five were formed from two separate sets of dots shown side by side, with a set of five always appearing on the left and the remainder on the right. All stimuli were presented using Pyschtoolbox (http://psychtoolbox.org/; Brainard, 1997; Pelli, 1997) and were projected ∼50 cm from the subject’s eye. Numerals and symbols subtended between 2° and 4° while dot sets subtended between 6° and 10°.

During each task, subjects viewed 240 trials, 120 of each trial type. All conditions contained an equal number of trials in which the set of dots matched or did not match the preceding numbers, while the two-word list condition additionally divided the matching trials equally among those that matched the first numeral and those that matched the second numeral. No trial contained repeated numerals, and so dot sets in the addition task ranged from three to nine while sets of size one to five were used in the list task. During each condition, each of the five numerals was used in the critical position an equal amount of times, half in matching and half in non-matching trials. Trial and stimuli lists were randomized and constructed separately for each subject, however within each experimental run, the initial and critical stimuli pairings were held constant between tasks. Thus, from the beginning of the trial until the end of the critical stimulus, subjects saw the same visual material in both tasks.

#### Procedure

During the experiment, subjects performed two separate blocks of trials, one of each task. Overall order of tasks was counterbalanced across all subjects. Before the experiment, subjects practiced their first task outside of the MEG room. Though subjects were made aware of the existence of a second task at this time, no specific instructions regarding the second task were given before the completion of the first task. Instructions and practice for this second task were then given following the completion of the first task, while subjects were in the machine.

During each trial, the initial fixation cross was shown for 300 ms while numeral and non-numeral stimuli were shown for 400 ms. Each stimulus was followed by a 300-ms blank screen. Sets of dots appeared at the end of each trial and remained onscreen until the subject made a decision. Subsequent trials began after a blank screen was shown for a variable amount of time, which followed a normal distribution with a mean of 500 ms and a standard deviation of 100 ms. The recording lasted ∼40 min.

#### Data acquisition and analysis

Magnetoencephalography data were recorded and preprocessed in exactly the same manner as in the pictorial experiment, resulting again in a distributed minimum norm estimate for each condition average for each subject. In the present experiment, two subjects failed to meet the preliminary requirements and were excluded from further analysis. The logic of the data analysis was also the same as before. A targeted ROI analysis served as our primary means to determine potential combinatorial effects, and this was then supported by a full-brain comparison. The only differences between the two sets of analyses were that, first, in the present experiment, instead of the lMFG, we investigated activity that localized to the IPS, and second, we employed the full interaction cluster test from Bemis and Pylkkänen (2011, 2012) during the ROI analysis in order to match the full two-task design. The IPS ROI was drawn to encompass a wide cortical region, in this case the entire horizontal IPS. As discussed above, this area has been widely implicated in tasks requiring numerical computations compared to the simple viewing of numerals (Dehaene et al., 2003), and thus we expect more activity in this region during the two-numeral addition condition compared to the corresponding control conditions. Though hypotheses regarding functional hemispheric asymmetries within this region have been put forward (Chochon et al., 1999), we chose to maintain simplicity in the present analysis and use a single symmetric bilateral IPS ROI.

The cluster test that we used to analyze ROI activity was identical in general to that applied to the pictorial data and identical in all respects to that applied to the linguistic data from Bemis and Pylkkänen (2011). The only difference between the two-condition test used in the pictorial experiment and the four-condition test in the current analysis was the specific test statistic used. As described in our previous studies, for the two-task design we expect combinatorial activity not only to exhibit an interaction between the two conditions in the two tasks, but we expect this interaction to have a specific form. Combinatorial mechanisms should elicit greater activity in the two-element combinatorial condition (addition in this case) compared to the matched one-element condition, while we expect no such difference in activity between the conditions of the control list task. Thus, the test statistic for the cluster test is calculated by first identifying contiguous points for which the interaction between the two factors, task and number of elements, reaches a given threshold, set as before at *p* = 0.30 as computed by a 2 × 2 repeated-measures ANOVA. Then, a paired samples *t*-test is performed within each task, between the one and two-element conditions, at each time point in the cluster and the absolute value of *t*-statistic of the control task is subtracted from the *t*-statistic of the combinatorial task. Thus, the test statistic tracks the extent to which the data matches our predicted combinatorial profile. The remainder of the test is as before, with the *p*-value of the observed statistic calculated against 10,000 permutations of the original data. Follow-up cluster tests and supplemental full-brain analyses were performed within each task and were identical in all respects to those used in the pictorial experiment.

### Results

#### Behavioral results

**Figure 6:**
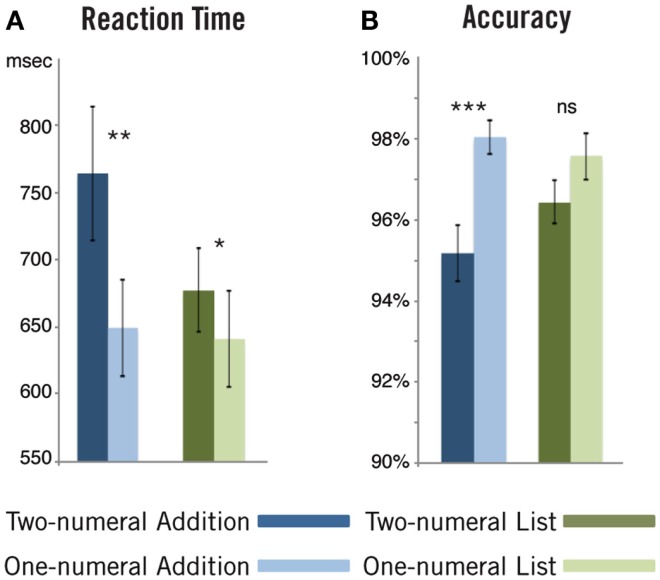
**Mathematical behavioral results**. Contrary to our previous linguistic paradigm, we found that subjects performed significantly worse on two-element combinatorial trials (the two-numeral addition condition in this case). Responses were both significantly slower **(A)** and less accurate **(B)** in this condition compared to the corresponding one-numeral control. We observed the same pattern of results in the List task, though to a lesser degree. ns, Non-significant; *p < 0.05; **p < 0.01; ***p < 0.001.

Reaction time and accuracy data were submitted to a 2 × 2 repeated-measures ANOVA with Task (addition, list) and Number of numerals (one, two) as factors (see Figure 6). We found a significant interaction for accuracy [*F*(1, 17) = 6.50, *p* = 0.02]. Though subjects were more accurate on one-numeral decisions compared to two-numeral decisions in both tasks, the interaction was driven by a larger difference in accuracy within the addition task [*p* < 0.0001; *t*(17) = 5.42; paired samples *t*-test, two-tailed; two-numerals: *M* = 95.2%, SD = 2.9%; one-numeral: *M* = 98.0%, SD = 1.8%] compared to the list task [*p* = 0.096; *t*(17) = 1.76; paired samples *t*-test, two-tailed; two-numerals: *M* = 96.4%, SD = 2.3%; one-numeral: *M* = 97.6%, SD = 2.5%].

For reaction time, we again found a significant interaction between Task and Number of numerals [*F*(1, 17) = 9.47, *p* = 0.0068]. In this case, subjects responded more quickly on one-numeral trials compared to two-numeral trials in both tasks, with the interaction again driven by a greater difference within the addition task [*p* = 0.0012; *t*(17) = 3.89; paired samples *t*-test, two-tailed; two-numerals: *M* = 765 ms, SD = 212 ms; one-numeral: *M* = 650 ms, SD = 153 ms] compared to the list task [*p* = 0.015; *t*(17) = 2.70; paired samples *t*-test, two-tailed; two-numerals: two-numerals: *M* = 678 ms, SD = 134 ms; one-numeral: *M* = 641 ms, SD = 152 ms].

These results conform largely to intuition and demonstrate that trials with two-numerals were harder than those with one, and that this difference was greater during the addition task. Beyond intuition, past studies find both slower and less accurate responses to simple calculation problems relative to non-calculation comparison responses (Chochon et al., 1999). However, while these behavioral results are not unexpected, it is worth noting that in our previous linguistic results two-element composition trials were actually *easier* than their one-element counterparts, and we found this result a well in the combinatorial pictorial condition. Thus, within the behavioral results we already find a divergence between simple mathematical calculation and basic linguistic combination.

#### ROI results

**Figure 7:**
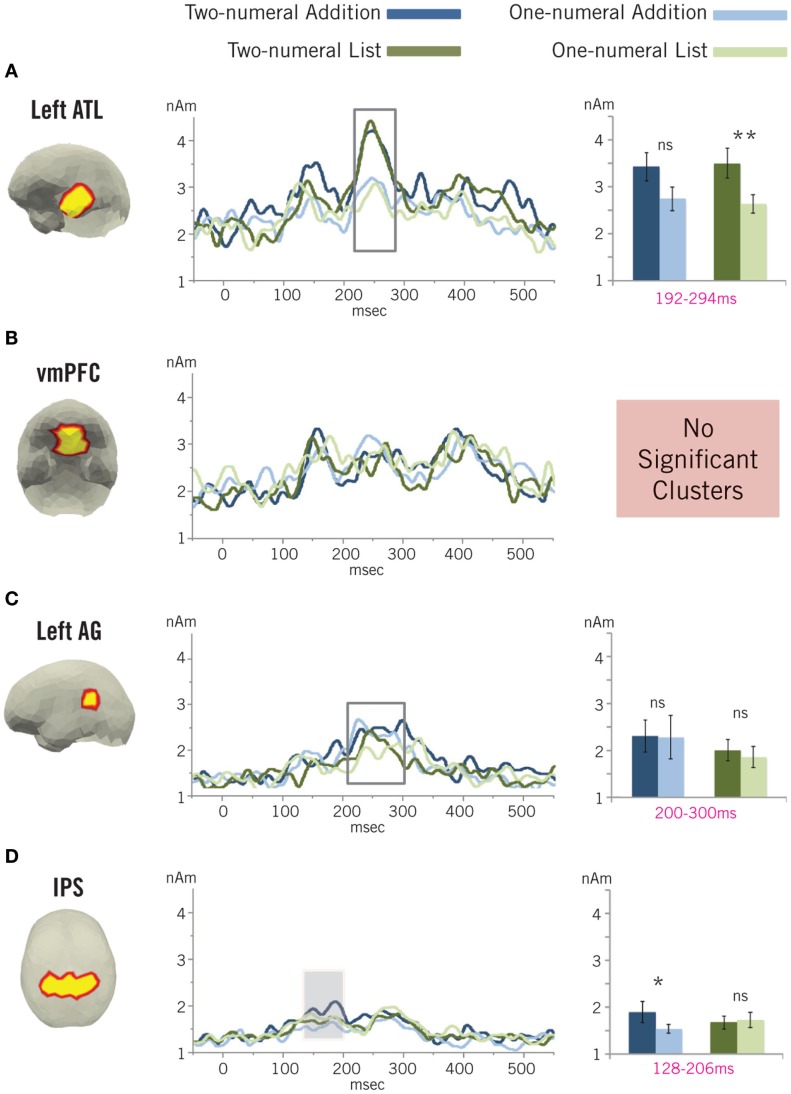
**Mathematical ROI results**. Localized activity is again shown for the three linguistic ROIs – the left ATL **(A)**, vmPFC **(B)**, and left AG **(C)** – and one ROI – the IPS **(D)** – intended to capture canonical effects related to mathematical processing. The shaded region denotes a significant cluster of increased activity in the IPS during basic addition, as identified by a permutation test (Maris and Oostenveld, 2007). The boxed region in the left ATL ROI denotes a significant main effect cluster for which activity was greater in both two-numeral conditions compared to their corresponding one-numeral control. The boxed region in the left AG ROI denotes a period of increased activity across all conditions. This activity, however, was not significantly different between any of the four conditions. ns, Non-significant; *p < 0.05; **p < 0.01.

Figure 7 shows the activity profiles for the four ROIs during processing of the critical stimuli. Only activity localized to the IPS ROI exhibited a significant addition-related effect. In this ROI, we identified a significant interaction cluster from 128 to 206 ms (*p* = 0.0147; 10,000 permutations) with greater activity in the two-numeral addition condition (*M* = 1.78 nAm, SD = 1.14 nAm) compared to its one-numeral control (*M* = 1.34 nAm, SD = 0.49 nAm), and no difference between the two-numeral (*M* = 1.51 nAm, SD = 0.74 nAm) and one-numeral (*M* = 1.57 nAm, SD = 0.84 nAm) list conditions. *Post hoc* cluster tests within each task revealed a matching, nearly significant cluster of increased activity in the IPS during two-numeral compared to one-numeral trials in the addition task from 131 to 209 ms, *p* = 0.0516 (10,000) and found no significant differences within the list task at any point (all clusters *p* > 0.70; 10,000 permutations).

Activity localized to the vmPFC ROI exhibited no significant effects at any point for any comparison (all interaction clusters, *p* > 0.60; all within-task clusters, *p* > 0.80; 10,000 permutations). Similarly, no significant effects were identified for activity localized to the lAG ROI at any point during the processing of the critical stimuli (all interaction clusters, *p* > 0.40; all within-task clusters, *p* > 0.40; 10,000 permutations). It is worth noting, however, that activity in this latter region exhibited a clear peak at ∼200–300 ms, though this component appears to not be differentiated across conditions. To follow-up on this impression, we submitted averaged activity from 200 to 300 ms that localized to the lAG to a 2 × 2 repeated-measures ANOVA with Task (addition, list) and Number of numerals (one, two) as factors. This exploratory analysis revealed no hint of an interaction [*F*(1, 17) = 0.049, *p* = 0.83] or any effect of number of numerals [*F*(1, 17) = 0.09, *p* = 0.76], and only a trend toward an effect of task [*F*(1, 17) = 2.42, *p* = 0.14] driven by increased activity in the addition task (two-numerals: *M* = 2.48 nAm, SD = 1.66 nAm; one-numeral: *M* = 2.45 nAm, SD = 2.26 nAm) compared to the list task (two-numerals: *M* = 2.13 nAm, SD = 1.11 nAm; one-numeral: *M* = 1.96 nAm, SD = 1.07 nAm).

Within the lATL ROI, we found no significant clusters of addition-related activity, with only a slight hint of such an effect late in the epoch from 467 to 489 ms (*p* = 0.1790; 10,000 permutations). There are clear peaks, however, present in both two-numeral conditions at ∼200–300 ms. To explore these increases, we performed an additional cluster test designed to identify main effects within the two tasks by summing the *t*-statistics from both tasks, instead of subtracting the list *t*-statistic from the addition value. This test identified a significant main effect cluster from 192 to 294 ms (*p* = 0.0064; 10,000 permutations) with increased activity in two-numeral conditions (addition: *M* = 3.76 nAm, SD = 1.46 nAm; list: *M* = 2.97 nAm, SD = 1.21 nAm) compared to their paired one-numeral controls (addition: *M* = 3.84 nAm, SD = 1.55 nAm; list: *M* = 2.85 nAm, SD = 0.94 nAm). Follow-up tests within each task indicated that this effect was stronger within the list task than the addition task. In the former, we found a significant cluster of increased two-numeral activity from 206 to 307 ms (*p* = 0.0123; 10,000 permutations), while in the latter, we only identified a marginal effect from 223 to 282 ms (*p* = 0.0905; 10,000 permutations). The late cluster of increased addition activity identified by the interaction test was also relatively weak according to the follow-up within-task test (446–493 ms; *p* = 0.1157; 10,000 permutations).

#### Full-brain results

**Figure 8:**
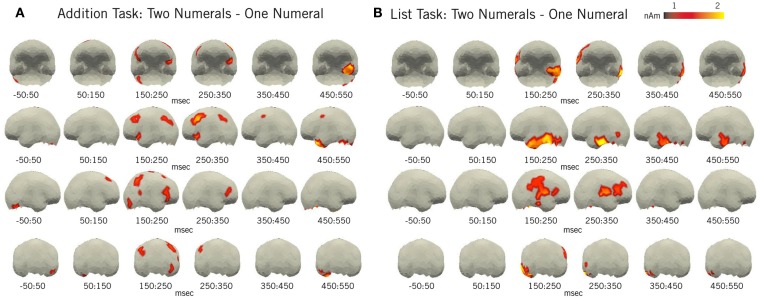
**Mathematical full-brain results**. Plotted regions denote the difference in average amplitude between the two-numeral and one-numeral conditions for both the **(A)** addition task and **(B)** list task, for all space-time regions in which two-numeral activity was reliably greater than one-numeral activity (by at least 1 nAm and *p* < 0.05, paired samples *t*-test) for at least 5 ms over five spatial neighbors. For clarity, non-cortical sources have been removed. Results in the addition task conform to our IPS ROI analysis and show a clear effect in this region at ∼150–300 ms. With respect to the main effect of number of numerals identified in lATL ROI, however, we found that, despite the similarity in ROI activity time courses, the effects near this region in each task are actually quite distinct, differing in location not only to each other but also to the effect observed in the previous linguistic study. Increased activity can also be observed over large parts of the frontal cortex during both tasks.

The supplemental full-brain analyses (Figure 8) support our IPS ROI results and show a robust early effect from 150 to 250 ms in both the left and right IPS during basic addition. No similar increase is seen within the list task comparison. Similarly, in accordance with our ROI results, we found no effects localized to either the vmPFC or lAG in the full-brain analysis.

Outside of the predefined ROIs, however, we found large regions of increased activity in the two-numeral conditions in both tasks. Within the addition task, there were early increases from 150 to 350 ms in the bilateral prefrontal cortex, with this activity appearing more dorsally in the left hemisphere and ventrally in the right. Within the list task, clear increases can be seen for much of the epoch (∼200–500 ms) in both temporal lobes. This activity localizes to the inferior, medial temporal lobe in the left hemisphere, while in the right hemisphere, activity spreads across a large anterior, dorsal region of the temporal lobe from 150 to 250 ms moving anteriorly into the prefrontal cortex at ∼250–350 ms. This pattern of results is not unexpected, especially in the list task, as we found increased activity in roughly the same regions – left inferior medial temporal cortex and right prefrontal cortex – in our linguistic list task (Bemis and Pylkkänen, 2011). Both regions have also been implicated in working memory tasks by other work (Petrides et al., 1993; Courtney et al., 1996; Wei et al., 2004). Within the addition task, the most prominent effect occurred in the left dorso-lateral prefrontal cortex; a finding consistent with past studies associating this region with basic mathematical computation (Dehaene et al., 2003).

The temporal lobe effects are more interesting, especially as related to our previous lATL ROI analysis. In the list task, while there is clear increased activity in the lATL at 150–250 ms, the central locus of this effect is posterior to the lATL ROI, within the medial temporal cortex. Conversely, in the addition task, the effect at ∼200 ms in proximity to the lATL occurs much more anteriorly than in the list task centering almost in the ventral inferior frontal gyrus. Only the late effect at ∼450 ms in the addition task appears to occur primarily in the lATL ROI. Thus, the full-brain analysis suggests that the main effect of number of numerals observed for activity localized to the lATL ROI may in fact actually reflect activity generated in two separate cortical regions during the two tasks.

### Discussion

In this experiment, we investigated the neural overlap between basic mathematical and linguistic processing by measuring activity generated during the simple addition of two small numerals and comparing the results to those observed previously during the composition of an adjective and a noun. As in the linguistic design, subjects completed both a combinatorial and non-combinatorial matching task, each of which contained two-element and one-element trials. In this case, however, the combinatorial task required subjects to judge whether a given number of dots accurately reflected the addition of the preceding numerical stimuli while the non-combinatorial task required subjects to judge if the following set of dots matched any of the preceding numerals. Consistent with many previous investigations into mathematical processing (Chochon et al., 1999; Dehaene et al., 2003, 2004; Roitman et al., 2012), we identified significantly increased activity associated with basic addition within the IPS. In the ROIs associated with combinatorial linguistic processing, however, we found no effects unambiguously associated with basic addition. We observed no significant differences in any comparison for activity localized to the vmPFC or lAG in the present manipulation. In the lATL, we observed both a significant main effect of numbers, with increased activity in both two-number conditions irrespective of task, and a relatively short, late increase during addition, at ∼450 ms, that failed to approach significance in any of our tests.

Thus, in general, we found little evidence that activity generated during basic linguistic composition reflects combinatorial processes also in operation during simple addition. This result is consistent with the hypothesis that our previously observed linguistic combinatorial effects reflect compositional mechanisms that are less broad than a general transformation from multiple inputs to a single output or the association of multiple cues with a memory trace. Further, this findings supports recent studies suggesting a clear functional distinction between mathematical and linguistic combinatorial processing (Martín-Loeches et al., 2006; Friedrich and Friederici, 2009; Fedorenko et al., 2011). The only clear combinatorial effect that we identified was instead localized to the IPS, in accordance with a wide range of previous neuromathematical results (Chochon et al., 1999; Dehaene et al., 2003).

We did, however, observe several effects that suggest the involvement of linguistic processing during the mathematical manipulation more generally, though these effects did not dissociate between tasks. We observed robust activity in the lAG for all conditions and a strong main effect of number of numerals in the lATL with increased activity in both two-number conditions compared to their paired one-numeral controls. At a broad level, these results are in agreement with much past evidence suggesting that linguistic representations play a prominent role within mathematical tasks but that this role is not combinatoric in nature but rather involves the storage and retrieval of linguistically encoded facts (Dehaene et al., 2003). While this hypothesis is roughly consistent with our observation of increased activity in the lAG throughout all tasks, possibly reflecting the linguistic coding of all numeric stimuli to some degree (Fulbright et al., 2000), the results from the lATL paint a somewhat more complex picture. The main effect revealed by the ROI analysis suggests a linguistic process that is modulated by the number of numeric elements in the trial. It should be noted that, although our previous paradigms revealed an interaction in lATL activity between task and number of words, in each case there also appeared to be increased activity during the two-word list condition as well compared to its paired one-word control (Bemis and Pylkkänen, 2011, 2012). Thus, since the lATL has been implicated in single word processing as well as combinatorial operations (Rogers et al., 2006; Patterson et al., 2007), the present finding may echo these more obscured linguistic results and reflect processing related to a single word, or verbally encoded numeral in this case, that is subject to interference from the preceding stimulus, as is the case for many lexical operations (Lau et al., 2008). This interpretation, however, must be tempered by the findings of the full-brain analysis that indicate that the apparent shared activity observed in the lATL ROI may actually originate from distinct cortical locations in the two tasks. In the addition task, increased activity in the two-numeral condition localized to the extreme anterior temporal pole while in the list task activity reflected in the lATL analysis was concentrated primarily in the middle and posterior temporal cortex. This finding raises the possibility that these apparently shared effects actually reflect disparate cognitive processes such as either the retrieval of specific facts (Rogers et al., 2006) in the addition task (or combinatorial processing, of course) and working memory processes (Martin and Chao, 2001) in the list task. On a more methodological note, the tension observed here between the ROI and full-brain analyses suggests that it may be inappropriate to lump entire cortical areas such as the lATL into a single ROI (cf. Fedorenko et al., 2011). Both neuroanatomical (Ding et al., 2009) and functional connectivity studies (Simmons et al., 2010) suggest that this region displays a large degree of heterogeneity and is thus likely to participate in a wide variety of cognitive functions. Thus, although the spatial resolution of MEG makes fined-grain claims regarding localization difficult – a limitation more severe in the lATL than other cortical regions (Hillebrand and Barnes, 2002) – the present results suggest that it may be fruitful to parcellate large cortical regions, such as the lATL, into smaller pieces for analysis. Localization accuracy may then be improved with the concurrent use of structural MRIs, when available (Dale et al., 2000).

Finally, it must be noted, of course, that while we found no clear evidence supporting the shared use of combinatorial linguistic mechanisms during addition, the interpretation of any null result must always be approached cautiously. It may be the case that the analysis, paradigm, signal to noise ratio, or many other factors might have limited the power of the experiment. In the present study, for example, while we found no significant effects in any of our linguistic ROIs accompanying basic addition, we did find a weak effect in the lATL at ∼450 ms that exhibited the expected addition-related activity. It might be the case that this effect reflects a shared combinatorial mechanism between language and math, perhaps related to the construction of generalized structural relationship between elements, which both domains embody to some degree. However, the extreme statistical weakness of this effect, along with the failure of previous investigations to uncover evidence of such shared neural activity (Friedrich and Friederici, 2009), prevents any strong conclusion to be drawn. Further, our current paradigm did prove powerful enough to detect a significant effect of addition in the IPS, in accordance with many past studies showing increased activity in this region during mathematical computation relative to controls (Chochon et al., 1999; Dehaene et al., 2003). Though the time interval of the observed effect is relatively early (∼100–200 ms), increased activity during numerical processing has previously been found in this general time window using both EEG (Dehaene, 1996) and intercranial recordings (Allison et al., 1994). Thus, this result not only confirms the power of the present paradigm to probe minimal mathematical computations, but also supports past evidence that the IPS plays an integral role in even the most basic mathematical operations.

## General Discussion

**Figure 9:**
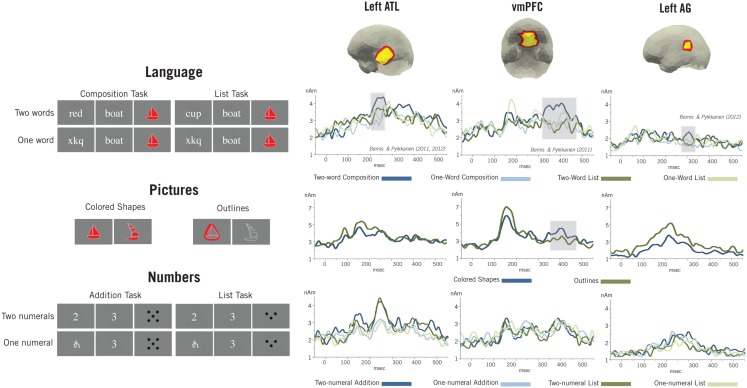
**Combined linguistic ROI results**. Activity generated during all tasks is shown for the three ROIs implicated in basic linguistic composition. Shaded regions denote significant clusters of increased activity during combinatorial conditions, as identified by a permutation test (Maris and Oostenveld, 2007). We observed no increased activity during basic addition in any of the ROIs. Combinatorial processing in the non-linguistic pictorial task evoked increased activity in the vmPFC, but not in either the left ATL or left AG.

The question of how language relates to cognition more generally has deep roots (Darwin, 1871). While several past neurocognitive investigations have probed this relationship in broad terms and found evidence for shared processing between relatively complex linguistic and non-linguistic tasks (Coulson et al., 1998; January et al., 2009; Rodd et al., 2010), the present study represents a first investigation into the extent to which basic combinatorial neural mechanisms operate outside of the language domain. Our results (summarized in Figure 9) indicate a partial overlap between combinatorial neural effects observed during the processing of basic adjective-noun combinations (Bemis and Pylkkänen, 2011, 2012) and those observed during conceptual combination elicited by non-linguistic stimuli in the present experiment. Specifically, we observed increased activity localized to the vmPFC during both types of combination at a similar time, while no corresponding effects were observed in either the lATL or lAG at any time. This result suggests that basic linguistic composition relies in part on a domain-general combinatorial operation, potentially in the service of constructing complex semantic representations. A test of more generalized combination using a mathematical paradigm that structurally paralleled our linguistic tasks indicated very little shared processing between basic addition and basic linguistic composition. Though not definitive, this result indicates that the combinatorial effects previously associated during linguistic composition do not reflect generalized combinatorial operations, such as producing a single representation from two distinct inputs or general memory processes that may relate two separate elements to a singular representation, and instead suggest that these effects are indicative of more specialized operations, such as the encoding of semantic or syntactic relationships between elements.

In a broad sense, this delineation between linguistic and non-linguistic regions is consistent with previous results. The vmPFC has been primarily implicated in non-linguistic tasks (Northoff et al., 2000; Sabbagh et al., 2004; Bayless et al., 2006), but not always (Mar, 2004), while the lATL and lAG have been linked most strongly to linguistic processes (Friederici et al., 2000; Humphries et al., 2001; Pallier et al., 2011), but not always (Gorno-Tempini and Price, 2001). Specifically, however, our results bring into focus the fact that delineating regions or processes as either “linguistic” or “non-linguistic” may be difficult at best and misleading at worst, as our findings suggest that even the most basic combinatorial linguistic mechanisms can be engaged by non-linguistic stimuli. At the present time the exact functional nature of the mechanisms subserved by these regions, either inside of or outside of language, remains unknown. Our past studies on adjective-noun combinations (Bemis and Pylkkänen, 2011, 2012) manipulated the presence or absence of composition as a whole and so did not have the ability to apportion different types of combination, such as the construction of syntactic structural relationships or the establishment of semantic conceptual relationships, to any particular region. The results of the present study are clearly most consistent with a conceptual, semantic role for the vmPFC during composition, as no explicitly syntactic structures are required during the pictorially evoked combination. This hypothesis is in accordance with past work implicating the vmPFC during the processing of linguistic expressions that require increased semantic processing relative to syntactically similar controls (Pylkkänen and McElree, 2007; Brennan and Pylkkänen, 2008). The lAG and the lATL, on the other hand, have previously been associated with syntactic processing, especially the lATL, which has been shown to correlate with measures of syntactic complexity during natural story comprehension (Brennan et al., 2012) and is suppressed during syntactic adaptation (Noppeney and Price, 2004). Both of these regions, however, have also been heavily implicated in semantic tasks as well (Price, 2000; Rogers et al., 2006). Thus, it is possible that one or both of these regions work in concert with the mechanism reflected by vmPFC activity to support operations involved in the semantic composition of linguistic expressions. Future work must now target these operations individually in order to tease apart the differential contributions of these regions and processes to linguistic, and non-linguistic, combination.

In sum, the results of the present study serve to situate the core combinatorial operations of language within cognition more broadly and provide evidence that at least certain facets of basic linguistic composition rely on neural mechanisms that can extend beyond the linguistic domain. Though these results are promising and demonstrate an ability to measure neural activity related to basic combinatorial processing across multiple domains, the present study constitutes merely the beginnings of this investigation. Further results are now needed from both within language and across additional domains in order to more completely delineate the functional role of basic linguistic combinatorial mechanisms and continue to chart the boundary between language and cognition.

## Conflict of Interest Statement

The authors declare that the research was conducted in the absence of any commercial or financial relationships that could be construed as a potential conflict of interest.
